# Complete response of sunitinib therapy for renal cell cancer recurrence in the native kidney after renal transplantation: a case report

**DOI:** 10.1186/1756-0500-7-526

**Published:** 2014-08-13

**Authors:** Fumiya Hongo, Masakatsu Oishi, Takashi Ueda, Yasuyuki Naitoh, Terukazu Nakamura, Yoshio Naya, Kazumi Kamoi, Koji Okihara, Tsuneharu Miki

**Affiliations:** Department of Urology, Kyoto Prefectural University of Medicine, 465 Kajii-cho Kamigyo-ku, Kyoto, 602-8566 Japan

**Keywords:** Complete response, Native kidney, Neoadjuvant therapy, Presurgical therapy, Renal cell cancer, Renal transplantation, Sunitinib, Tumor thrombus

## Abstract

**Background:**

No case report has yet shown that sunitinib therapy for the postoperative recurrence of renal cancer in a native kidney after renal transplantation can achieve complete response (CR).

**Case presentation:**

A tumor was detected in the right native kidney of a 35-year-old Japanese male 10 years after renal transplantation. A tumor thrombus that reached the atrium was detected, which suggested cT3cN0M0. Because of the risk of perioperative complications, preoperative therapy with sunitinib was selected and 8 courses were administered. The size of the primary tumor was reduced by 33%, while that of the tumor thrombus was decreased by 39.5%.

Right nephrectomy and removal of the tumor thrombus were then performed. Contrast-enhanced computed tomography (CT) four months after surgery suggested local relapse. Sunitinib was administered for 9 months, which led to complete response (CR).

**Conclusions:**

This study presented the case of sunitinib therapy for renal cancer in the native kidney after renal transplantation. The therapeutic efficacy and safety for such cases should be discussed.

## Background

The usefulness of preoperative drug therapy for improving the safety of surgery or preserving kidney function in patients with progressive renal cancer has been reported with the introduction of molecule-targeting drugs, [1], but this has yet to be confirmed. Sunitinib, a multitargeting tyrosine kinase inhibitor (TKI), has been established as a first-line therapy for metastatic clear cell renal cell carcinoma (cc RCC) [2] and was administered in the present case.

Complete response (CR) has been reported previously in patients treated with sunitinib or sorafenib, but was rare at less than 1% [3, 4]. No case report has yet showed that sunitinib therapy for the postoperative recurrence of renal cancer in a native kidney after renal transplantation can achieve CR.

## Case presentation

A 35-year-old Japanese male presented with abdominal pain.

**Figure 1:**
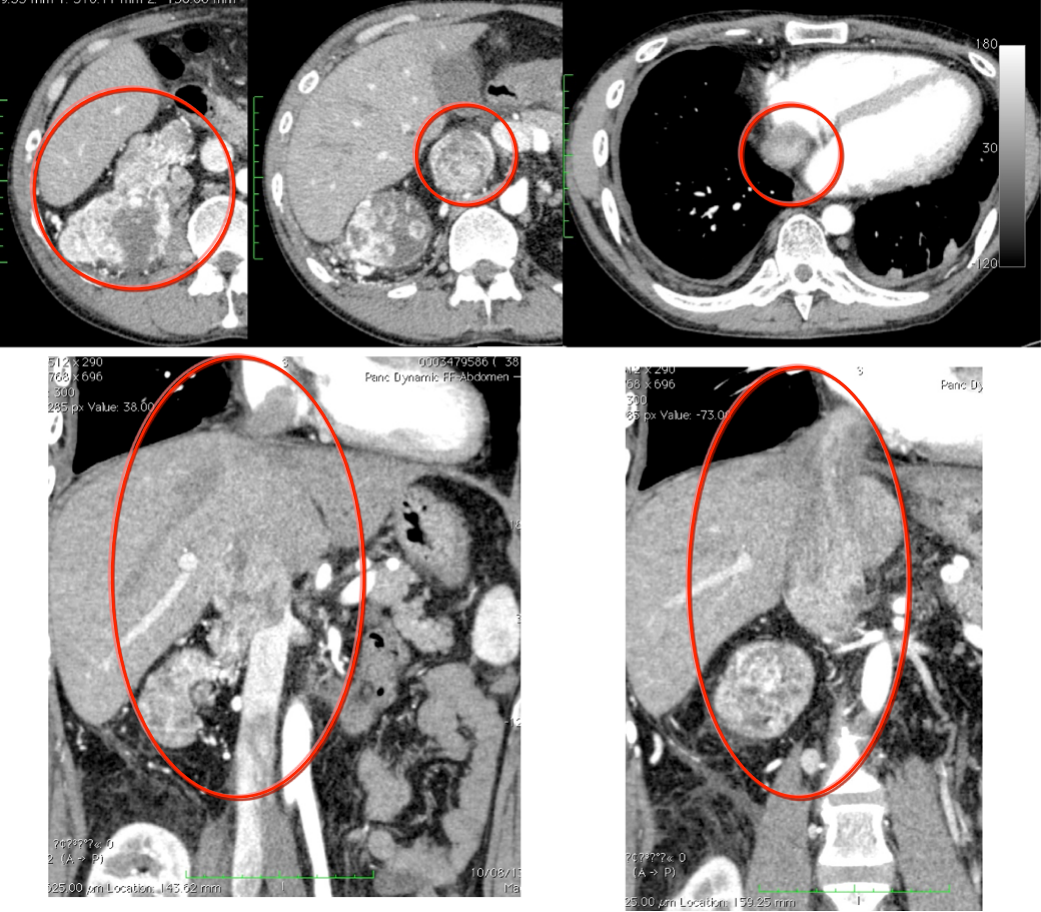
**Computed tomography scan before preoperative sunitinib therapy.** The level of tumor thrombus was evaluated as IV according to Novic’s classification. The red circle indicates the tumor thrombus.

Computed tomography (CT) revealed a right renal tumor with a venous to arterial thrombus (Figure 1). No remarkable invasion in the peripheral organs or metastasis was observed. The stage of the tumor was determined to be cT3N0M0. The level of the tumor embolism was evaluated as IV based on Novic’s classification. The eastern cooperative oncology group- performance status (ECOG-PS) was evaluated as 1. The patient had a medical history of renal transplantation at 26 years of age and immunoglonblin A (IgA) nephropathy, and was being treated with the immunosuppressants tacrolimus and prednisolone.

After reviewing whether surgery should be performed promptly or drug therapy should initially be conducted, the risk of perioperative complications was explained to the patient and his family. Preoperative drug therapy with sunitinib was subsequently selected. The risk according to the Memorial Sloan Kettering Cancer Center (MSKCC) scale was regarded as intermediate (hemoglobin and time from the diagnosis to treatment).

Eight courses of sunitinib therapy were administered. Because the patient had previously undergone renal transplantation, the initial dose of sunitinib administered was 25 mg. This dose was gradually increased while monitoring the patient for adverse events: 1^st^ to 2^nd^ courses, 25 mg; 3^rd^ to 4^th^ courses, 37.5 mg; 5^th^ to 7^th^ courses, 50 mg; and 8^th^ course, 37.5 mg. Edema was exacerbated during the 2 weeks of discontinuation; therefore, the discontinuation period was established as 7 to 10 days from the 4^th^ course. The relative dose intensity was 79% in all courses.

**Figure 2:**
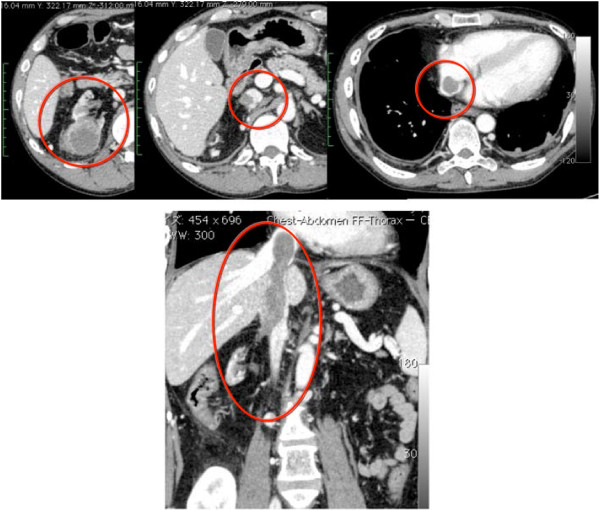
**Computed tomography scan after 10 months of preoperative sunitinib therapy.** The level of tumor thrombus was evaluated as IV according to Novic’s classification; there were no changes from before sunitinib therapy. However, the size of the primary tumor was reduced by 33%, and that of the tumor thrombus decreased by 39.5%. The red circle indicates the primary tumor and thrombus.

After the 1^st^ course had been completed, CT revealed that the sizes of the primary focus and tumor thrombus were decreased by 13% and 17%, respectively (Response evaluation criteria in solid tumors [RECIST] ver 1.1). Sunitinib therapy was conducted for 10 months. Although no changes were observed in the level of the tumor thrombus (IV), the size of the primary tumor decreased by 33%, while that of the tumor thrombus was reduced by 39.5% (Figure 2). Edema (G2), fever (G1), general malaise (G2), and an increase in creatinine level (3.71 mg/dl, G2) were observed as adverse events.

Surgery was performed using the following procedures: an L-shaped dermal incision, liver mobilization, securing the right ureter/renal vein, and ligation of the renal artery.

Peripheral inferior vena cava (IVC) blood flow was blocked by cardiopulmonary bypass. The IVC to the right atrial region was incised to extirpate the right atrial tumor. Adhesion was noted between the tumor embolus and vascular wall. The right kidney and tumor embolus were extirpated as a mass. The IVC wall was partially and simultaneously resected.

The operation time was 17 hours and 43 minutes. The volume of intraoperative blood lost was 9,807 cc, while that of blood loss in the presence of a Cell Saver® was 23,578 cc. Regarding blood transfusions, 130 units of red blood cells (RBC), 80 units of fresh frozen plasma (FFP), and 20 units of platelets (Plt) were required.

Histopathologically, clear cell carcinoma with a sarcomatoid component (less than 10%) (pT3c, G2 = G1 > G3, INFc, v1, ly0, ig, fc1) was suggested. A viable cancer cell nest with vitrification and necrosis was observed in the tumor embolus.

Right subclavian arterial hemorrhage (post operative day (POD) 14, Clavien Grade IIIa), abdominal wound infection (POD 35, Grade IIIb), and central venous catheter infection (POD 89, Grade II) occurred as perioperative complications. The patient was discharged on the 113^th^ postoperative day.

### Postoperative course

**Figure 3:**
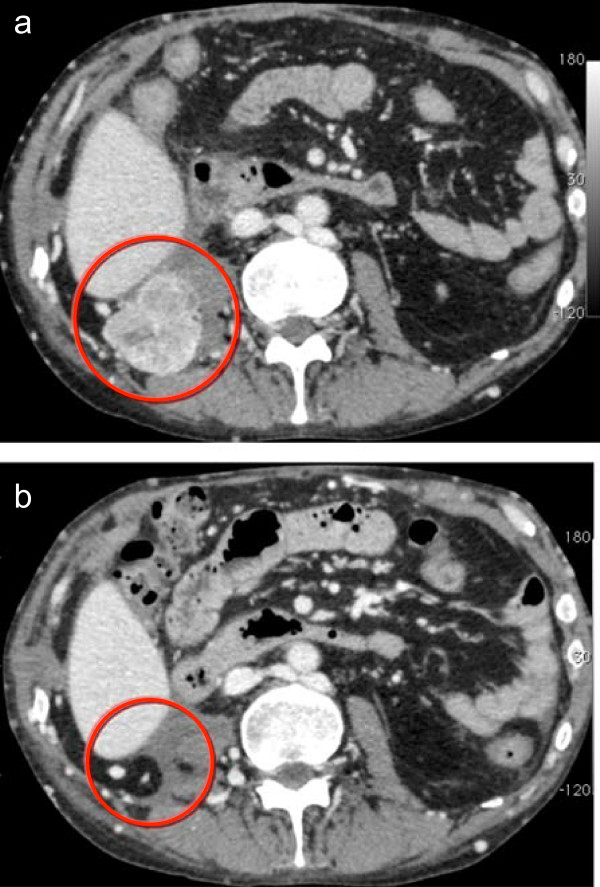
**Postoperative computed tomography scan.** A complete response was achieved after the 7^th^ course of sunitinib therapy for local relapse. Four months after surgery, a computed tomography scan showed that the tumor had increased to 45 mm **(a)**. The administration of sunitinib was started. After 9 months of therapy, a complete response was achieved **(b)**. The red circle indicates the local recurrent tumor.

CT revealed a recurrent tumor (measuring 20 mm) in the right retroperitoneal floor 1 month after surgery, which had increased to 45 mm at 4 months after surgery (Figure 3a). A partial response was achieved after 3 months of therapy, as shown by a 73% reduction in the size of the tumor. CT showed a CR after the administration of sunitinib for 9 months (Figure 3b). An additional course was subsequently administered, and the treatment was completed after 11 months. No relapse or metastasis as observed during the 2-year follow-up following completion of the administration of sunitinib (interval from the start of treatment: 3 years), and CR was maintained.

## Discussions

The preoperative administration of drugs was not commonly performed previously. However, the introduction of molecule-targeting drugs for metastatic renal cancer has markedly changed the treatment algorithm [5]. TKI was shown to reduce the size of tumors, which suggested the efficacy of preoperative administration. Schrader *et al*. [6] reviewed 33 patients who were administered treatments preoperatively, and showed a reduction in the size of the tumor embolus in most cases, which facilitated surgery. However, preoperative administration was not beneficial for all patients. A retrospective, large-scale study was performed to examine the vena cava tumor thrombus-minimizing effects of molecule-targeting drugs [7]. As a prospective study, a phase II study involving 30 patients preoperatively administered sunitinib was performed. The median rate of the decrease observed in the primary tumor (renal clear cell carcinoma) size was 28% (absolute reduction: 1.7 cm). Of these patients, the primary endpoint of being able to undergo nephrectomy after preoperative sunitinib therapy was achieved in 13 patients (45%) [8].

TKIs such as sunitinib and sorafenib may represent treatment options because they are not contraindicated in patients with a transplant under immunosuppressive therapy [9]. Tumor reductions of approximately 40% and 20% have been achieved previously with sunitinib and sorafenib, respectively [10–12]. Therefore, we chose sunitinib as the therapy option.

In the present case, no changes were observed in the level of the tumor thrombus after preoperative administration; however, its size was reduced by 33.9%.

Relapse was detected after surgery. However, drug therapy rapidly reduced the size of the tumor, leading to CR. The proportion of patients with CR achieved by administration of a TKI such as sunitinib is reportedly 1% or less [4]. Another study showed that CR was achieved by the administration of TKIs (sequential sunitinib-sorafenib) for local relapse after nephrectomy [13]. Because cancer is a mass of diverse clones, the degree of vascular endothelial growth factor (VEGF) dependency may vary. In the present case, differences were detected in CT findings between the recurrent and primary tumors; the former remained unchanged, while changes were observed in the latter. Therefore, the recurrent tumor may have been a group of highly VEGF-dependent cells with relatively similar characteristics to the primary tumor. Therefore, CR may have been associated with TKI treatment. The appropriateness of discontinuing the administration of sunitinib after achieving CR has not yet been fully discussed [14]. In this study, the drug continued to be administered for a short term after CR was achieved, and was only discontinued after it had been confirmed that CR was maintained, and, in the absence of relapse, had not resumed.

To the best of our knowledge, the safe use and effectiveness of sorafenib, but not sunitinib has already been described for immunosuppressed renal transplant recipients with advanced RCC [15].

Surgery for RCC with a tumor thrombus in the IVC, especially Levels III to IV, is challenging. Previous case reports, including the present case, have not suggested the usefulness of preoperative administration.

## Conclusions

A case of pre- and postsurgical sunitinib treatment for a renal transplant recipient is reported herein. The neoadjuvant sunitinib treatment did not decrease the tumor thrombus with arterial extension. On the other hand, sunitinib therapy led to CR for local relapse after surgery. We described only one case and did not have enough data and reviewed literatures. The therapeutic efficacy and safety as in such cases should be studied.

## Consent

Written informed consent was obtained from the patient for publication of this Case Report and any accompanying images. A copy of the written consent is available for review by the Editor-in-Chief of this journal.
